# Marketing authorization of COVID-19 vaccines across UK, EU, and the US: fact-checking and the implications for future research

**DOI:** 10.1186/s40545-021-00400-0

**Published:** 2021-12-24

**Authors:** Nasir Abbas, Zaheer-Ud-Din Babar

**Affiliations:** Department of Pharmacy, University of Huddersfield, Huddersfield, HD1 3DH UK

**Keywords:** Marketing authorization, COVID-19 vaccines, EU, US, UK

## Abstract

While having access to safe and efficient vaccines is essential for eradicating the COVID-19 pandemic, gaining marketing authorisation is a critical step in enabling and speeding this process. On December 2, 2020, the United Kingdom became the first country to approve the first COVID-19 vaccine. This commentary aims to provide a quick overview of the UK’s COVID-19 vaccine authorization process and compare it to that of the EU and the US. While the UK, EU, and US expedited the COVID-19 vaccine approval process, regulatory authorities did not appear to cut corners in their approval of the Pfizer COVID-19 vaccine, as evidenced by their decisions to switch emergency use authorization to full authorization in the US and to renew conditional/temporary use authorization in the EU and UK, respectively. There is an opportunity to conduct a thorough investigation into and comparison of the filed dossiers, as well as the robustness of the evaluation process for the approval of COVID-19 vaccines.

## Introduction

United Kingdom (UK) is the first country, where Pfizer-BioNTech COVID-19 Vaccine was approved on 02 December 2020 [1]. This has allowed UK population to access this much needed vaccine few weeks ahead of anyone else in the world [2]. This commentary aims to provide a quick summary of authorization process for the COVID-19 vaccines in the UK and compares the process with the EU and the US. Several COVID-19 vaccines have now been approved for use in humans [3]. In this commentary we have reviewed the authorization and review process of Pfizer-BioNTech COVID-19 Vaccine (mRNA Vaccine BNT162b2 concentrate for solution for injection) now branded as Comirnaty^®^. This is first approved COVID-19 vaccine and has been approved for use in 112 countries so far, and 46 trials in 21 countries have been conducted [4].

Ensuring access to new medicines including vaccines is a global concern [3, 5]. While access to safe and effective vaccinations is key to eliminating the COVID-19 pandemic, obtaining marketing authorization is a crucial step in enabling and expediting this process. This Authorization process for new medicines including vaccines is long and complex and is considered a barrier to early access. However, during this pandemic renowned regulatory bodies across the world have adapted/devised most efficient mechanisms to authorize the COVID-19 vaccines by reducing the review time from several months to few weeks resulting in faster access to vaccines. Before the end of transition period (31 December 2020) of the UK’s withdrawal from the EU, licensing for innovative medicinal product in the European Union including UK would have been granted only by the European Medicines Agency (EMA). Nevertheless, UK modified the legislation in 2020 to enable temporary authorization for emergency use during the pandemic. This enabled UK Medicines and Healthcare products Regulatory Agency (MHRA) to authorize and approve it without getting an approval from EMA [6].

### Temporary use authorization of COVID-19 Vaccines in the United Kingdom

In UK Pfizer-BioNTech COVID-19 Vaccine was primarily granted a temporary authorization for emergency use. Temporary use authorizations allow for the supply of an unlicensed pharmaceutical product in response to specific sorts of public health threats, such as the suspected or verified spread of pathogenic agents [7]. In the UK, for the review of “Pfizer-BioNTech COVID-19 Vaccine” a request for temporary use authorization was made to Medicines and Healthcare products Regulatory Agency (MHRA). The evaluation process started on 1st October and an approval was granted on 2nd December 2020. This was a rolling data submission as the pharmaceutical company was allowed to submit the data related to product efficacy and safety in batches as it become available [8].

After the UK’s approval of the Pfizer/BioNTech vaccine, the FDA and the EMA followed suit and issued emergency use authorizations for the same vaccine, including for pregnant women [9, 10]. UK’s temporary use authorisation once granted lasts for 1 year as pharmaceutical companies are required to fulfil specific obligations under this. The standard marketing authorization is granted once complete data package is available [11]. Authorization in UK was renewed on 02 December 2021 [12].

### Conditional marketing authorization of COVID-19 Vaccines in the European Union

In EU Pfizer-BioNTech COVID-19 Vaccine was primarily approved through a procedure called conditional marketing authorisation. Conditional marketing authorization is one of the EU's regulatory mechanisms for promoting early access to pharmaceuticals that meet an unmet medical need, such as the current pandemic [13]. In the EU, review of the request for conditional market authorization for “Pfizer-BioNTech COVID-19 Vaccine” to European Medicines Agency (EMA) started on 5th October and an approval was granted on 21 December 2020. Though EMA used rolling data submission but the overall review process in EU took few more weeks as compared to the UK. This resulted in the EU approval a little later [14]. Similar to MHRA temporary use authorization, EMA conditional marketing authorization is initially granted for 1 year which can either be renewed before the end of the year or pharmaceutical company can provide more data and request for standard marketing authorization [15]. After reviewing updated quality, safety, and effectiveness data and verifying the positive benefit–risk balance, the EMA has recommended on October 14, 2021 that the conditional marketing authorization for Pfizer-BioNTech COVID-19 Vaccine be renewed [16].

### Emergency use authorization of COVID-19 vaccines in the United States

In the US Pfizer-BioNTech COVID-19 Vaccine was primarily approved through a procedure called Emergency Use Authorization (EUA). This procedure is for simplifying the provision of medical countermeasures, including vaccines, during public health disasters like as the current COVID-19 pandemic. When certain statutory criteria are met, such as that there are no adequate, approved, and available alternatives, FDA may allow the use of unapproved medical products, or unapproved uses of approved medical products, in an emergency to diagnose, treat, or prevent serious or life-threatening diseases or conditions [17]. In the US, the review of the request for Emergency use authorization of “Pfizer-BioNTech COVID-19 Vaccine” to US Food and Drug Administration (FDA), started on 20th November and an approval was granted on 11th December 2020. Though review in US started few weeks later but the overall review time was less when compared with the UK and EU [18]. Delay in start of review in US was because of FDA advice to pharmaceutical companies for not requesting Emergency use authorization until 50% of the patients had completed 2 month follow-up after administration of vaccines [19]. FDA has expanded the emergency use authorization for Pfizer-BioNTech COVID-19 Vaccine to full authorization on 23 Aug 2021. While millions of people have already gotten COVID-19 vaccinations successfully under emergency use authorization, FDA’s full authorization of this vaccine may give some people more confidence to get vaccinated [20].

### Summary of COVID-19 vaccine approval processes in the UK, EU, and the US

While UK, EU and US have also expedited the COVID-19 vaccine approval process, regulatory authorities in these countries have made no compromise on quality, efficacy & safety standards [6, 21]. A key difference between the expedited procedures (emergency use authorization/temporary use authorization/conditional marketing authorization) used for COVID-19 vaccines and standard licencing procedures is the length of study participants’ follow-up to evaluate the duration of protection and if there were any rare adverse events down the road. The average follow-up period used for COVID-19 vaccine procedures is 2 months, but standard licencing procedures require 6 months [22]. It is important to note that COVID-19 vaccines, SINOPHARM in China, and Sputnik V in Russia have been approved for use even before the completion of phase 3 trials. Genetic vaccines are new, and long-term safety testing is required for identifying potentially contraindicated groups of persons, such as those with a history of blood disorders, thrombocytopenia (past or present), or pre-existing immunological diseases [23]. It is also important to note that following initial approval, pharmaceutical companies agreed with regulatory authorities on various measures, including monitoring systems, to ensure that any safety concerns are identified and evaluated in a timely manner [20].

## Conclusions

UK, EU, and US regulatory authorities did not appear to have compromised the evaluation standards to approve the Pfizer COVID-19 vaccine and it is primarily evident by regulatory authorities’ decisions to switch emergency use authorization to full authorization in US and renewal of conditional/temporary use authorization in EU and UK, respectively. During the COVID-19 vaccines approval process, unprecedented level of acceleration of regulatory processes and interactions between regulatory authorities and pharmaceutical companies have contributed to delivering the vaccines to market sooner. These learning could be leveraged not only in dealing future pandemic but also to optimize regulatory strategy for important treatments with unmet medical need. There is an opportunity to do a detailed study to investigate and compare the submitted dossiers and robustness of the evaluation process for approval of the COVID-19 vaccines in general and particularly Pfizer COVID-19 vaccine.

## References

[1] UK medicines regulator gives approval for first UK COVID-19 vaccine—GOV.UK [Internet]. [cited 2021 Jan 31]. Available from: https://www.gov.uk/government/news/uk-medicines-regulator-gives-approval-for-first-uk-covid-19-vaccine.

[2] Ledford H, Cyranoski D, Van Noorden R. The UK has approved a COVID vaccine—here’s what scientists now want to know. Nature. NLM (Medline); 2020. p. 205–6. 10.1038/d41586-020-03441-8 33288887

[3] Wouters OJ, Shadlen KC, Salcher-Konrad M, Pollard AJ, Larson HJ, Teerawattananon Y, et al. Challenges in ensuring global access to COVID-19 vaccines: production, affordability, allocation, and deployment. Health Policy. 2021. 10.1016/S0140-6736(21)00306-8 PMC7906643 33587887

[4] WHO—COVID19 Vaccine Tracker [Internet]. [cited 2021 Dec 13]. Available from: https://covid19.trackvaccines.org/agency/who/.

[5] Abbas N, Hasan SS, Curley L, Babar Z-U-D. Access to medicines—a systematic review of the literature. Res Social Adm Pharm. 2020;16. 10.1016/j.sapharm.2019.12.009 31839584

[6] Vagnoni C, Barber S. Regulatory approval of COVID-19 vaccines in the UK [Internet]. [cited 2021 Jan 31]. Available from: https://post.parliament.uk/regulatory-approval-of-covid-19-vaccines-in-the-uk/.

[7] The mechanics of medicines regulation—shining a spotlight on the MHRA vaccine approval decision | Brodies LLP [Internet]. [cited 2021 Dec 13]. Available from: https://brodies.com/insights/healthcare-and-life-sciences/the-mechanics-of-medicines-regulation-shining-a-spotlight-on-the-mhra-vaccine-approval-decision/.

[8] Public Assessment Report Authorisation for Temporary Supply COVID-19 mRNA Vaccine BNT162b2 (BNT162b2 RNA) concentrate for solution for injection.

[9] Mahase E. Vaccinating the UK: how the covid vaccine was approved, and other questions answered. The BMJ. BMJ Publishing Group; 2020. 10.1136/bmj.m4759 33298577

[10] Forman R, Shah S, Jeurissen P, Jit M, Mossialos E (2021). COVID-19 vaccine challenges: what have we learned so far and what remains to be done?. Health Policy.

[11] The mechanics of medicines regulation—shining a spotlight on the MHRA vaccine approval decision | Brodies LLP [Internet]. [cited 2021 Jan 31]. Available from: https://brodies.com/insights/healthcare-and-life-sciences/the-mechanics-of-medicines-regulation-shining-a-spotlight-on-the-mhra-vaccine-approval-decision/.

[12] Summary of Product Characteristics for COVID-19 Vaccine Pfizer/BioNTech—GOV.UK [Internet]. [cited 2021 Dec 13]. Available from: https://www.gov.uk/government/publications/regulatory-approval-of-pfizer-biontech-vaccine-for-covid-19/summary-of-product-characteristics-for-covid-19-vaccine-pfizerbiontech.

[13] EMA recommends first COVID-19 vaccine for authorisation in the EU | European Medicines Agency [Internet]. [cited 2021 Dec 13]. Available from: https://www.ema.europa.eu/en/news/ema-recommends-first-covid-19-vaccine-authorisation-eu.

[14] CHMP. Committee for Medicinal Products for Human Use (CHMP) Assessment report Comirnaty Common name: COVID-19 mRNA vaccine (nucleoside-modified).

[15] Conditional marketing authorisation | European Medicines Agency [Internet]. [cited 2021 Jan 31]. Available from: https://www.ema.europa.eu/en/human-regulatory/marketing-authorisation/conditional-marketing-authorisation.

[16] CHMP. Committee for Medicinal Products for Human Use (CHMP) Assessment report on the annual renewal of the conditional marketing authorisation. [cited 2021 Dec 13]; Available from: www.ema.europa.eu/contact.

[17] Emergency Use Authorization for Vaccines Explained | FDA [Internet]. [cited 2021 Dec 13]. Available from: https://www.fda.gov/vaccines-blood-biologics/vaccines/emergency-use-authorization-vaccines-explained.

[18] Administration D. Emergency Use Authorization (EUA) for an Unapproved Product Review Memorandum Identifying Information Application Type EUA (Event-driven EUA request) Application Number 27034 Sponsor Pfizer, Inc., on behalf of Pfizer and BioNTech Submission Date. 2020.

[19] How key decisions slowed FDA’s review of Covid-19 vaccine [Internet]. [cited 2021 Jan 31]. Available from: https://www.statnews.com/2020/12/04/how-key-decisions-slowed-fdas-review-of-covid-19-vaccine-but-also-gave-it-important-data/.

[20] FDA Approves First COVID-19 Vaccine | FDA [Internet]. [cited 2021 Dec 13]. Available from: https://www.fda.gov/news-events/press-announcements/fda-approves-first-covid-19-vaccine.

[21] Fast-forward: Will the speed of COVID-19 vaccine development reset industry norms? | McKinsey [Internet]. [cited 2021 Dec 16]. Available from: https://www.mckinsey.com/industries/life-sciences/our-insights/fast-forward-will-the-speed-of-covid-19-vaccine-development-reset-industry-norms.

[22] Emergency Use Authorization and FDA approval, vaccine fact check | wusa9.com [Internet]. [cited 2021 Dec 13]. Available from: https://www.wusa9.com/article/news/verify/emergency-use-authorization-fda-approval-vaccines-fact-check/65-7391e595-cee0-4a00-8468-194a6e0a21a4.

[23] Merchant HA (2021). CoViD vaccines and thrombotic events: EMA issued warning to patients and healthcare professionals. J Pharm Policy Pract.

